# Chemical Fingerprint of Floral Nectar in Apple (*Malus* sp.) Cultivars Grown in Norway

**DOI:** 10.3390/antiox15010103

**Published:** 2026-01-13

**Authors:** Milica Fotirić Akšić, Mirjana Pešić, Ilinka Pećinar, Mihajlo Jakanovski, Danijel Milinčić, Aleksandar Kostić, Marko Kitanović, Uroš Gašić, Dragana Dabić Zagorac, Dušanka Milojković Opsenica, Mekjell Meland

**Affiliations:** 1 Faculty of Agriculture, University of Belgrade, Nemanjina 6, 11000 Belgrade, Serbia; fotiric@agrif.bg.ac.rs (M.F.A.); mpesic@agrif.bg.ac.rs (M.P.); ilinka@agrif.bg.ac.rs (I.P.); danijel.milincic@agrif.bg.ac.rs (D.M.); akostic@agrif.bg.ac.rs (A.K.); marko.kitanovic@agrif.bg.ac.rs (M.K.); 2 Innovative Centre of the Faculty of Chemistry, University of Belgrade, Studentski Trg 12-16, 11000 Belgrade, Serbia; jakanovski@chem.bg.ac.rs (M.J.); dusankam@chem.bg.ac.rs (D.M.O.); 3 Institute for Biological Research “Siniša Stanković”, University of Belgrade, Bulevar Despota Stefana 142, 11060 Belgrade, Serbia; uros.gasic@ibiss.bg.ac.rs; 4 Faculty of Chemistry, University of Belgrade, Studentski Trg 12-16, 11000 Belgrade, Serbia; naca10@gmail.com; 5 Department of Horticulture, NIBIO Ullensvang, Norwegian Institute of Bioeconomy Research, N-5781 Lofthus, Norway

**Keywords:** *Malus domestica*, Malus sylvestris, sugars, phenolic acids, flavonoids, antioxidant capacity, Raman

## Abstract

This study included the nectar of nine standard apple (*Malus* × *domestica*) cultivars (‘Red Aroma’, ‘Discovery’, ‘Summerred’, ‘Rubinstep’, ‘Elstar’, ‘Asfari’, ‘Eden’, ‘Fryd’, and ‘Katja’) and two crab apple (*Malus sylvestris*) cultivars (‘Dolgo’ and ‘Professor Sprenger’). The aim was to determine the diversity of chemical compounds in the floral nectar of the two different apple species and their cultivars. Chemical analysis identified five sugars, two sugar alcohols, two organic acids, forty phenolic compounds, and five phenylamides. The crab apples ‘Dolgo’ and ‘Professor Sprenger’, along with the commercial cultivar ‘Rubinstep’, had the highest levels of all three main sugars (glucose, sucrose, and fructose). The cultivar’s ‘Katja’ nectar had the highest level of total phenolic content (60.7 mg/100 g GAE), the nectar sample from ‘Dolgo’ exhibited the greatest ability to neutralise DPPH radicals (83.4 mg/100 g TE), and the ‘Dolgo’ (100.6 mg/100 g TE FW) and ‘Katja’ (72.1 mg/100 g TE FW) nectars proved to be the best reducing agents. Floral nectar from ‘Eden’ and ‘Fryd’ showed very high levels of isorhamnetin, 49.04 mg/kg and 50.83 mg/kg, respectively, while nectar from ‘Katja’ had the highest level of gentisic acid at 39.06 mg/kg. Besides being vital for insects, apple floral nectar is a significant reservoir of phenolic compounds and can be considered a “superfood” for the human diet.

## 1. Introduction

Apple (*Malus × domestica* Borkh.) are the most significant temperate fruit species globally with a total production of approximately 97.3 million tonnes. China produces about 49.6 million tonnes (almost 51% of global production), followed by Turkey (4.6 million tonnes) and the USA (4.1 million tonnes) [1]. This member of the Rosaceae family has one of the largest gene pools, with genotypes varying in ripening time, size, colour, taste, transportability, and many other traits. Due to its low rate of fruit deterioration and year-round storability, apples are one of the most popular snack fruits. In the northern hemisphere, the largest areas where apples are grown are within the latitude range of 25° N to 52° N. Apples are also grown beyond this range if the regions have a favourable climate and/or are warmed by water masses, such as in Scandinavia [2]. In Norway, the northernmost fruit tree-growing area in the world, the most common apple cultivars are ‘Discovery’, ‘Summerred’, ‘Red Gravenstein’, ‘Red Aroma’, and ‘Rubinstep’, while crab apples are mostly used as polliniser [3,4,5].

As with most *Rosaceous* species, the apple exhibits gametophytic self-incompatibility, so it is necessary to plant compatible cultivars alongside the main cultivar to ensure fertilisation, seed, and fruit set [6]. Additionally, coexisting cultivars must have overlapping flowering periods to promote effective pollen transfer and facilitate inter-cultivar pollination [7]. The stamens of apple flowers allow nectar-gathering bees to obtain nectar by pushing their tongues between the filaments without touching the anthers or stigma [6]. Honey bees collect both pollen and nectar from the flowers. Pollen collectors are attracted to flowers that have just started shedding pollen and show lower constancy to one cultivar, while nectar collectors prefer older flowers with a higher volume of nectar from a particular cultivar [8,9].

Floral nectar is a crucial link in the interaction between insect-pollinated plants and their pollinators [10]. This interaction between insects and apple trees is mutually beneficial: insects assist in fertilisation and receive nectar in return. Nectar is a primary energy source for many insects, including bees, which mix it with pollen and use it in their nutrition [11]. Floral nectar is secreted through the nectaries, and its composition and concentration depend on the flower’s age, blooming stage, and various ecological factors [12]. Nectar secretion may also be influenced by abiotic factors such as water availability, temperature, solar radiation, soil type, and vapour pressure [13]. Moderately elevated temperatures may increase nectar secretion, but significantly higher temperatures reduce it [14]. Meanwhile, nectar sugar concentration is generally less variable and less affected by temperature than nectar volume [15].

Nectar is primarily composed of water, reaching 80%, and sugars (mainly glucose, fructose, and sucrose, as well as minor sugars such as mannose, arabinose, xylose, maltose, melibiose, raffinose, melezitose, stachyose, and sorbitol) [16]. The sugar composition and nectar concentration may also vary between species, cultivars, rootstocks, blooming stages, and flower ages [12]. Baker and Baker [17] and Hendriksma et al. [18] investigated the relationship between nectar chemical composition and pollinators, noting that honey bees prefer nectar with a high sugar concentration and sucrose-rich content. Based on the concentration of sucrose (S) and its ratio to fructose (F) and glucose (G), four types of nectar are distinguished: hexose-dominant S/(G + F) < 0.1, hexose-rich S/(G + F) = 0.1–0.49, sucrose-rich S/(G + F) = 0.5–0.99, and sucrose-dominant S/(G + F) > 1. If a plant species produces sucrose-rich nectar, the volumes are low but more concentrated, while hexose-rich nectars are usually abundant but diluted due to osmolarity and the movement of water from plant tissue into the nectar [19]. The level of sucrose has decreased throughout the evolution of plant families, with high proportions in ancient families (*Papaveraceae* and *Ranunculaceae*) and lower levels in more recently evolved families such as *Asteraceae* and *Violaceae,* with *Rosaceae* having intermediate levels [20]. Mujagic et al. [21] concluded that bees do not perceive nectar as sweet unless the sucrose ratio reaches 3% of the total sugar content. However, the sucrose thresholds of individual bees depend on many exogenous and endogenous factors, such as weather, hive condition, available pollen, the bee’s foraging specialisation, and genetic background [22].

Besides sugars, nectar contains small amounts of other organic and inorganic compounds such as free amino acids (which serve as a nitrogen source for pollinators), polyphenols, alkaloids, enzymes (which maintain nectar homeostasis), vitamins, organic acids, metal ions (K, Na, Ca, and Mg), proteins, lipids, coumarins, pigments, volatiles, and saponins [16,19]. Nicolson [16] reported that the most important phenols in floral nectar are quercetin, kaempferol, luteolin, naringenin, cinnamic acid, vanillic acid, and caffeic acid. Secondary metabolites, especially polyphenolics (chlorogenic acid, phloretin, naringenin, quercetin, catechin, and hydroquinone) and proteins (nectarins), in floral nectar have been associated with antimicrobial defensive functions; therefore, it is understandable why various kinds of honey have different antibiotic properties [12,23,24].

From an ecological perspective, secondary metabolites in nectar play a crucial role by attracting pollinators or repelling nectar thieves, thereby shaping plant–pollinator interactions [25]. Polyphenols in nectar also act as signalling molecules to predators and parasitoids and may serve as visual cues for insects due to their fluorescent properties [26,27]. The presence of amino acids in nectar influences its taste, with bees showing a preference for proline and phenylalanine [28,29].

Floral volatile emissions serve as advertisements to attract pollinators and facilitate reproduction. Honey bees can use floral volatiles to distinguish subtle differences among flowers, based on both the intensity of the floral scent and the ratios of volatile organic compounds in a complex mixture [30]. Among these volatiles, asarone, hexahydrofarnesyl acetone, and phytol are present in apple nectar [31].

To date, nectar has been analysed in many pome and stone fruit species of the *Rosaceae* family, such as the apple [31,32,33,34], pear [35,36], quince [37], plum [38], peach and apricot [35], and sweet [31] and sour cherry [39]. In the present study, we analysed the chemical fingerprint of nectar secreted by different apple (*Malus* sp.) cultivars grown under the ecological conditions of western Norway. The objective was to characterise the sugar and polyphenolic profiles, antioxidant capacity, and protein level (with Raman spectroscopy) in nectar and to demonstrate the diversity of chemical compounds in different apple species and cultivars.

## 2. Materials and Methods

### 2.1. Ecologic Conditions

The temperature in Ullensvang during the growing season (May–September) averaged 13.4 °C over the last 30 years, with July as the warmest month, averaging 16.1 °C. The annual accumulated precipitation is 1705 mm, and the average for the growing season is 459 mm [40]. Soil factors in Norwegian orchards vary, ranging from sandy soils with high rainfall influenced by the mild climate in the fjord area on the west coast of Norway to soils with finer texture and lower rainfall in the south-eastern part of the country [41].

### 2.2. Plant Material and Nectar Collection

The nectar of 11 different apple cultivars (Table 1) was collected from an intensive orchard in Lofthus, West Norway (municipality of Ullensvang, latitude 60°19′8.03″ N, longitude 6°39′14.31″ E). The orchard’s design and maintenance are described in Fotirić Akšić et al. [42]. Each cultivar was represented by at least 15 trees, with ‘Dolgo’ and ‘Professor Sprenger’ as pole trees. On six trees per cultivar, four branches were selected and isolated with tulle nets. The branches with flowers used for nectar analysis were isolated during bud opening, and nectar was collected over three to four consecutive days, starting from the full flowering stage, from several branches. Nectar collection was carried out in the morning (9–11 am), when nectar production is highest. The procedure for pollen collection is described in Fotirić Akšić et al. [36]. Data were gathered and further analysed over three repetitions, with the tables and figures presenting the average values.

### 2.3. Reagents and Standards

Sugar standards (glucose, fructose, sucrose, isomaltose, trehalose, sorbitol, and mannitol) were purchased from Supelco/Sigma-Aldrich (St. Louis, MO, USA). All aqueous solutions were prepared using ultrapure water (0.055 µS/cm) obtained by using the Thermo Fisher TKA MicroPure water purification system (Waltham, MA, USA).

### 2.4. Determination of Sugars and Sugar Alcohols by IC

A high-performance anion-exchange liquid chromatography system with pulsed amperometric detection was used to analyse sugars and sugar alcohols. Chromatographic measurement was performed using a Dionex ICS 3000 DP LC system (Dionex, Sunnyvale, CA, USA) equipped with a quaternary gradient pump and electrochemical detector, which consisted of Au as the working electrode and Ag/AgCl as the reference electrode, an autosampler (AS-DV), and Chromeleon software (Chromatography Workstation and Chromeleon 6.7 Chromatography Management Software). All separations were performed on a Carbo Pac PA100 column (4 × 250 mm (analytical) and 4 × 50 mm (guard); Dionex) thermostated to 30 °C. The mobile phase flow rate was 0.7 mL/min, and mobile phase composition was changed (gradient elution) during the analysis in the following order: −20–5 min = 15% 300 mM NaOH; 5–12 min = 15% 300 mM NaOH and 2% 500 mM NaOAc; 12–20 min = 15% 300 mM NaOH and 4% 500 mM NaOAc; 20–30 min = 20% 300 mM NaOH and 20% 500 mM NaOAc; rest to 100% is ultrapure water. Total analysis run time: 30 min [43].

### 2.5. UHPLC Q-ToF MS Analysis

Prior to analysis, nectar samples were made up to a volume of 5 mL with milliQ water, intensively vortexed, and passed through SPE cartridges (CLEAN-UPR, C18 extraction columns, Unendcapped-PKG50, UCT, Bristol, UK) to remove sugars and other unwanted compounds. The SPE cartridges were conditioned with acidified methanol and milliQ water. Finally, adsorbed phenolics were eluted with acidified methanol, filtered through 0.22 µm filters, and analysed by UHPLC Q-ToF MS.

The separation, identification, and characterisation of bioactive compounds of apple nectar was performed on an Agilent 1290 Infinity ultra-high-performance liquid chromatography (UHPLC) system coupled with a quadrupole time-of-flight mass spectrometer (6530C Q-ToF-MS) (Agilent Technologies, Inc., Santa Clara, CA, USA), using the same UHPLC method and Q-ToF operating parameters as previously described in detail by Kostić et al. [44]. For suspect screening, data-dependent acquisition (DDA) was used with the auto MS/MS acquisition mode (100–1700 *m/z*, scan rate of 1 spectra/s), with the fixed collision energy set to 30 eV. Agilent MassHunter software was used for instrument control and data analysis. The exact masses of the components were calculated using ChemDraw software (version 12.0, CambridgeSoft, Cambridge, MA, USA). Some of the identified phenolic compounds were quantified using available standards and their content was expressed in mg/kg nectar. Table S1 shows a list of phenolic compounds used for quantification and their equation parameters.

### 2.6. Proximate Phytochemical Composition Expressed as Total Phenolic Content

The determination of total phenolic content (TPC) in the nectar samples was performed as described in Fotirić Akšić et al. [45]. The results are expressed as mg/100 g gallic acid equivalents (GAEs), based on the fresh weight (FW) of the samples.

### 2.7. Antioxidant Activity Examination

The ability of the samples to exhibit antioxidant properties was investigated using three different assays (ferric-reducing power (FRP), ABTS^•+^, and DPPH^•^ quenching ability), as described in previous research [45]. All results obtained are expressed as Trolox equivalents (TEs), based on the FW of the samples.

### 2.8. Raman Spectral Acquisition

The Raman spectra were recorded with the Raman spectrometer XploRA (Horiba Jobin Yvon, Palaiseau, France), which is equipped with laser excitation at a wavelength of 532 nm and a grating with 1200 lines/mm. The device is equipped with an Olympus BX 41 microscope (Olympus, Tokyo, Japan) and a 50 LWD (Long Working Distance) objective (Olympus, Tokyo, Japan). The spectra were recorded using LabSpec 6.0 software (Horiba Jobin Yvon, France) with an exposure time of 5 s and by scanning the sample five times with a 100% filter. The calibration was checked using the 520.47 cm^−1^ line of silicon. The assignment of the main bands in the range from 200 to 1800 cm^−1^ was based on literature data.

### 2.9. Data Pre-Processing and Analysis

Principal component analysis (PCA), for the quantitative data obtained in this study, was performed using the PLS Toolbox software package for MATLAB (Version 7.12.0), Budapest, Hungary. Before applying PCA, all data were group-scaled prior to PCA. The singular value decomposition (SVD) algorithm was selected, along with a 0.95 confidence level for Q and Hotelling T2 limits to identify outliers.

The raw spectra obtained in Raman analysis were pre-processed the following methods: baseline correction with Savitzky–Golay filters with 7 points and smoothing of the spectra with a second-order polynomial function using the Spectragryph 1.2.14 software (Oberstdorf, GERMANY) [46]. Pre-processed spectra were subjected to Principal component analysis (PCA) with the PAST software [47].

## 3. Results and Discussions

For many *Rosaceae* species, including apple, nectar volume can be very small or viscous, making it difficult to collect sufficient quantities for comprehensive chemical analyses [7]. During dry and sunny days, the water in the nectar may evaporate, leaving behind a concentrated sugar residue or crystals which can be blown away by wind or washed off by rain [8]. In our study, nectar production followed a daily cycle, with flowers showing an early morning peak (producing most of their nectar between 9 and 11 am), followed by a rapid decline. In some cultivars, nectar removal was followed by either an increase or decrease in nectar production. These patterns were not easy to follow and it depended on pollinators and environmental factors.

### 3.1. Sugar Profile of Nectar

Most of the nutritional value of nectar comes from three simple sugars—sucrose and its component hexoses, glucose, and fructose. Nectar sugars originate from sucrose transported from the phloem to nectar tissue. Sucrose produced by photosynthesis may be temporarily stored as starch in the nectar and later degraded. Hydrolysis of sucrose should produce a 1:1 ratio of glucose to fructose, but an imbalance in fresh nectar indicates the involvement of other biochemical pathways [48].

In all apple nectar samples investigated, five sugars and two sugar alcohols (Table 2) were identified. The most abundant sugars in the nectar were fructose, glucose, and sucrose. Fructose content ranged from 0.21 (‘Fryd’) to 24.58 g/100 g (‘Rubinstep’). Glucose content ranged from 0.10 (‘Katja’) to 19.45 g/100 g (‘Rubinstep’), while sucrose content varied from 0.01 (‘Fryd’) to 5.41 g/100 g (‘Red Aroma’). In this experiment, fructose accounted for 31.88–92.23% of all sugars, glucose for 16.53–97.72%, and sucrose for 0.83–43.67%. Our results are consistent with those obtained in other studies [35,49]. Although nectars and their composition have been linked to the level of bee activity, in apple, nectar should contain appreciable amounts of all three sugars [35]. In our study, fructose was up to approximately twice as high as glucose, but glucose was up to approximately 46-fold higher than sucrose (‘Discovery’). Long ago, Baker [50] concluded that highly complex, bilaterally symmetrical flowers provide sucrose-rich nectars, while morphologically less complex (actinomorphic) flowers, such as apple, have hexose-rich nectar. Nicolson [48] also stated that deep and concealed flowers have sucrose-rich nectars while shallow flowers with more exposed nectaries, just like apple, tend to have hexose-rich nectars. The exception is ‘Asfari’, which had the highest level of sucrose compared to glucose and fructose. Besides ‘Red Aroma’, ‘Dolgo’ also had a high level of sucrose, which is very important since sucrose levels, rather than total sugar concentrations, are more influential on bee activity [51].

According to Baker and Baker’s [17] equation for nectar grouping, it can be concluded that ‘Discovery’, ‘Summerred’, and ‘Elstar’ had hexose-dominant nectar (S/(G + F) < 0.1) and ‘Asfari’ had sucrose-rich nectar (S/(G + F) = 0.5–0.99), while all others had hexose-rich nectar (S/(G + F) = 0.1–0.49). This supports the fact that the hydrolysis of sucrose to hexoses is more favourable at low temperatures, resulting in higher hexose proportions at high latitudes or altitudes [52]. Butterflies, moths, and long-tongued bees usually prefer sucrose-rich nectars, whereas short-tongued bees and flies prefer nectar rich in fructose and glucose [53,54]. Nagy Toth et al. [12] stated that in stone fruit, early-blooming cultivars produce sucrose-poor or sucrose-free nectar, while in later-blooming cultivars, the level of sucrose increases. In our case, this was also observed, as ‘Red Aroma’, one of the latest blooming cultivars studied, had the highest level of sucrose, while ‘Discovery’, the earliest, had the lowest sucrose levels.

Regarding minor sugars, isomaltose content ranged from 0.21 g/100 g (‘Rubinstep’) to 5.11 g/100 g (‘Dolgo’), while trehalose content ranged from 0.08 g/100 g (‘Asfari’) to 4.50 g/100 g (‘Professor Sprenger’). Isomaltose is more closely associated with honeydew than with nectar, which explains why honeydew honeys contained significantly higher levels of this sugar than blossom honeys [55]. The three cultivars with the highest levels of isomaltose also showed the highest resistance to economically important diseases (‘Dolgo’ is resistant to scab, rust, and mildew, with good resistance to fire blight; ‘Professor Sprenger’ has good resistance to fire blight and rust and moderate resistance to apple scab; and ‘Red Aroma’ is resistant to apple scab). Similar findings regarding high levels of isomaltose were observed in pear cultivars resistant to *Psylla* sp. [36].

Trehalose is another nectar component that enhances sweetness for insects and plays an important role in abiotic stress protection by serving as a protective barrier for proteins and cellular membranes [56].

According to Bieleski and Redgwell [57], although sorbitol is the major soluble carbohydrate in plants of the woody *Rosaceae*, the floral nectars contained virtually no sorbitol. In our study, it was the most abundant sugar alcohol, with content ranging from 0.04 g/100 g (‘Eden’) to 3.49 g/100 g (‘Dolgo’). Although sorbitol is a minor component in nectar, it is readily converted to other sugars by nectaries [45]. According to Fotirić Aksić et al. [36], as the levels of sorbitol, glucose, and fructose in pear nectar increased, resistance to *Psylla* sp. also increased.

The mannitol content ranged from 0.04 g/100 g (‘Asfari’) to 2.88 g/100 g (‘Professor Sprenger’). These results are expected, as carbohydrates for nectar production are mainly imported from the phloem solution, which consists primarily of sorbitol in members of the *Rosaceae* family, along with mannitol and oligosaccharides of the raffinose family [58,59].

The sum of the quantified sugars was approximately 17.5 times higher than the sugar alcohol content in the analysed nectar samples. The total amount of quantified sugars and sugar alcohols ranged from 0.51 g/100 g (‘Fryd’) to 50.84 g/100 g (‘Professor Sprenger’), corresponding to values obtained in other studies [35,49]. Our results differ from those of Meheriuk et al. [35], who found no appreciable differences among cultivars regarding nectar quality within a fruit species, which was not the case in our experiment. Such discrepancies may be due to the analysis of different apple genotypes and the environment of completely diverse microclimatic conditions. Besides this, the methods used for nectar collection may vary significantly between studies. In our experiment nectar extraction was performed directly with microcapillaries, while in Meheriuk et al. [35], nectar was collected by washing flowers with 80% alcohol.

### 3.2. UHPLC Q-ToF MS of Apple Nectar

Phenolics in floral nectar can attract pollinators, have detrimental effects on pollinators, be repellent to some visitors, defend against attack by herbivores and microorganisms, and serve as a guide for pollinators [60,61]. Phenolics increase the attractiveness of sucrose solution to honey bees, probably by adding odour to the solution, making it easier to locate [62].

Table 3 summarises all bioactive compounds in the floral nectar of different apple varieties identified by “untargeted” UHPLC Q-ToF MS analysis. In contrast to apple fruits, leaves, or floral pollen, which have already been extensively investigated [45,63,64], the bioactive compounds of apple nectar have hardly been analysed [31,65]. In addition, only a few studies have investigated the volatile or phenolic compounds of floral nectar from sour cherry [31,39], pumpkin [31], pear [36], açai (*Euterpe oleracea*) [66], linden [67], or some wild plant species [63]. In this study, a total of 47 compounds were tentatively identified. Phenolic compounds were the most abundant in the analysed nectar samples and were the most frequently confirmed (40 compounds), followed by phenylamides (5 compounds) and only 2 organic acids. As shown in Table 3, most of the identified compounds were found selectively in the analysed apple nectar samples, while only hydroxycinnamic acid amide tricoumaroyl spermidine and flavonoid kaempferol 3-*O*-rhamnoside, were found in all samples.

Phenolic acids were mostly detected as glycosides or as esters with quinic acid; only hydroxybenzoic acid, dihydroxybenzoic acid, gallic acid, and caffeic acid were confirmed as aglycones. In addition to the methyl and ethyl derivatives of gallic acid were also detected, which are known as methyl and ethyl gallate. All identified hydroxybenzoic acid glycosides (compounds **3**, **4**, **6**, and **11**) have previously been found and reported in apple leaves [64] and floral pollen (except compound **6**) [45]. Hydroxycinnamic acids (coumaric, caffeic, and ferulic acids) were mostly found as conjugates with quinic acid (compounds **13**, **16**, **18**, and **19**) and rarely as glycosides or aglycones. Caffeic acid was detected in all nectar samples, except those of ‘Rubinstep’ and ‘Dolgo’. In contrast the ethyl derivative of caffeic acid (ethyl caffeate) was only found in ‘Red Aroma’ nectar. This aspect could be justified considering that low concentrations or the absence of caffeic acid are preferred by pollinators, probably due to its repellent taste at high doses [68], and by the fact that ‘Rubinstep’, ‘Red Aroma’, and ‘Dolgo’ are considered excellent pollinisers in Norwegian apple orchards [69]. However, it was not possible to exclude the possibility that caffeic acid, after its synthesis, could be immediately converted into its derivatives (chlorogenic acid and *p*-coumaric acid), resulting in these two phenolic acids always being present at higher levels than caffeic acid itself and generally showing an opposite accumulation trend [70]. In our study, the level of chlorogenic acid was up to approximately seven times higher than that of caffeic acid.

Coumaroylquinic acid, caffeoylquinic acid, and/or feruloylquinic acid were found in most of the nectar samples analysed (see Table 3 and Table S2). This is expected, as these derivatives are characteristic compounds of apple fruit [71,72], leaves [63,64], and apple pollen [45]. Different isomers of coumaroylquinic acid and caffeoylquinic acid have also been reported in pear nectar [36]. Aesculetin was detected in all samples except in ‘Dolgo’ nectar, whereas aesculin was detected only in the ‘Elstar’ nectar. Aesculetin was previously reported in pear nectar samples [36].

In addition to phenolic acids, various flavonoids were also detected in the samples analysed, indicating a significant presence of these compounds in apple nectar, especially flavonol glycosides. Among the flavonols, the various kaempferol, quercetin, isorhamnetin, and syringetin glycosides were the most frequently detected. Kaempferol 3-*O*-rhamnoside was confirmed in all nectar samples, while kaempferol 3-*O*-pentoside was found only in ‘Red Aroma’, ‘Rubinstep’, ‘Eden’, and ‘Fryd’. The identified kaempferol and quercetin glycosides have previously been reported as typical compounds of apple fruits, peels, leaves, and pollen [45,72,73]. Compounds **35** and **36** were identified as isorhamnetin 3-*O*-(2″-*O*-rhamnosyl)hexoside and isorhamnetin 3-*O*-(2″-*O*-hexosyl)hexoside, respectively, while compound **38** was identified as isorhamnetin 3-O-(2″-hexosyl-6″-maloyl)hexoside. These isorhamnetin derivatives have already been reported in pear nectar [36] and floral apple pollen [45]. Syringetin aglycone and its glycosides (compounds **40** and **41**) have been previously detected in apple floral pollen as the predominant syringetin derivatives. Eriodictyol and taxifolin aglycones were selectively found in nectar samples (see Table 3 and Table S2). These compounds and their glycosides have already been reported in apple peel [63]. Dihydrochalcones are characteristic and the most frequently detected compounds in various apple samples [64,73]. The importance of phloridzin is evident from its use in the chemotaxonomic differentiation of rosaceous plant species [74] and in identifying fraudulent admixtures of apple juice with other fruit juices [75]. Within *M.* × *domestica*, the quantity of dihydrochalcones can vary depending on the tissue (pollen, branches, root bark, seeds, leaves, and immature and mature fruits), cultivar, developmental stage, sampling time, and external factors such as pathogen attack and in many cases is associated with disease resistance [76,77]. In this study, only two phloretin glycosides were identified, namely, phloretin 2′-*O*-hexoside (phloridzin) and phloretin 2′-*O*-(6″-*O*-hexosyl)hexoside, which were confirmed only in ‘Professor Sprenger’ nectar (compounds **44** and **45**), while they were absent from the other nectar samples.

The phenolic compounds for which standards were available were quantified (four phenolic acids and isorhamnetin) and are listed in Table 4. Dihydroxybenzoic acid (gentisic acid) was predominant in ‘Katja’ nectar (39.06 mg/kg), while its content was significantly lower in the other nectar samples (1.48–6.35 mg/kg). This compound in floral nectar plays a major role in defence against microbial attack, helping to protect the nectar and, by extension, the reproductive parts of the flower from spoilage [78]. Previously, Hagler and Buchmann [68] tested gentisic and caffeic acids in nectar and found that they were repellent at high concentrations but at low concentrations did not deter bees and even increased attractiveness.

Gallic acid was quantified only in ‘Asfari’ and ‘Fryd’ nectar samples. In some nectars, it can be oxidised by a nectar peroxidase into ellagic acid, which later, with iron, creates a dark pigment that serves as a visual attractant for pollination [79]. Bees consuming nectar with gallic acid can experience both positive (health benefits) and negative (reduced gland size) effects, highlighting the complex impact of phytochemicals on colony health [80].

Caffeic acid was quantified in the nectar samples from ‘Professor Sprenger’, ‘Asfari’, ‘Eden’, and ‘Fryd’ (0.87–2.54 mg/kg). It influences plant–pollinator interactions and defence mechanisms and can protect insects from parasites [81]. According to Bernklau et al. [82], gallic acid, together with caffeine (an alkaloid), *p*-coumaric acid (a phenolic acid), and kaempferol (a flavonol), significantly increases worker bee longevity and promotes pathogen tolerance. Caffeoylquinic acid (chlorogenic acid) was predominant in ‘Eden’ nectar (8.59 mg/kg), followed by ‘Asfari’, ‘Dolgo’, and ‘Fryd’ nectar samples, while in other samples it was present only in trace amounts or was absent. Chlorogenic acid is often the most abundant phenolic acid in nectar, especially in *Crataegus monogyna* [83]. It should be noted that caffeic acid, after its synthesis, may have been immediately converted into its derivatives (chlorogenic acid and *p*-coumaric acid), resulting in these two phenolic acids always being present at higher levels than caffeic acid itself and generally showing an opposite accumulation trend [70]. In our study, the level of chlorogenic acid was up to approximately seven-fold higher than that of caffeic acid.

Isorhamnetin is the only flavonoid detected in quantitative amounts, with the highest content in ‘Fryd’ (50.83 mg/kg) and ‘Eden’ (49.04 mg/kg) nectar samples, followed by ‘Asfari’ and ‘Professor Sprenger’, while its content was significantly lower in other nectar samples. As with other phenolic compounds, isorhamnetin in nectar can have antioxidant and antimicrobial properties, potentially helping to protect the flower against microbial attack and deterring some nectar robbers or herbivores [84]. Stanojević et al. [85] detected higher levels of isorhamnetin in organic acacia and chestnut honey compared to conventional counterparts.

Phenylamides are typical compounds in bee-collected pollen samples [44,86,87], whereas they have hardly been analysed in nectar samples so far, with the exception of *Euphorbia resinifera* floral cyathia [88]. In addition, our previous research has shown that floral apple pollen is a rich source of various putrescine and spermidine derivatives with coumaroyl, caffeoyl, and/or feruloyl moieties [45]. In this study, five putrescine and spermidine derivatives were confirmed in the nectar samples. These phenylamides were also previously reported in apple floral pollen [45], and the presence of pollen grains apparently contributes to their content in nectar samples. The phenylamide identified as *N*^1^*,N*^5^*,N*^10^-tri-coumaroyl spermidine was confirmed in all samples analysed, while other phenylamides were selectively present in nectar samples. All identified phenylamides were found only in ‘Red Aroma’, ‘Elstar’, and ‘Eden’ nectar samples.

### 3.3. Total Phenolic Content and Antioxidant Activity of Nectar Samples

**Figure 1:**
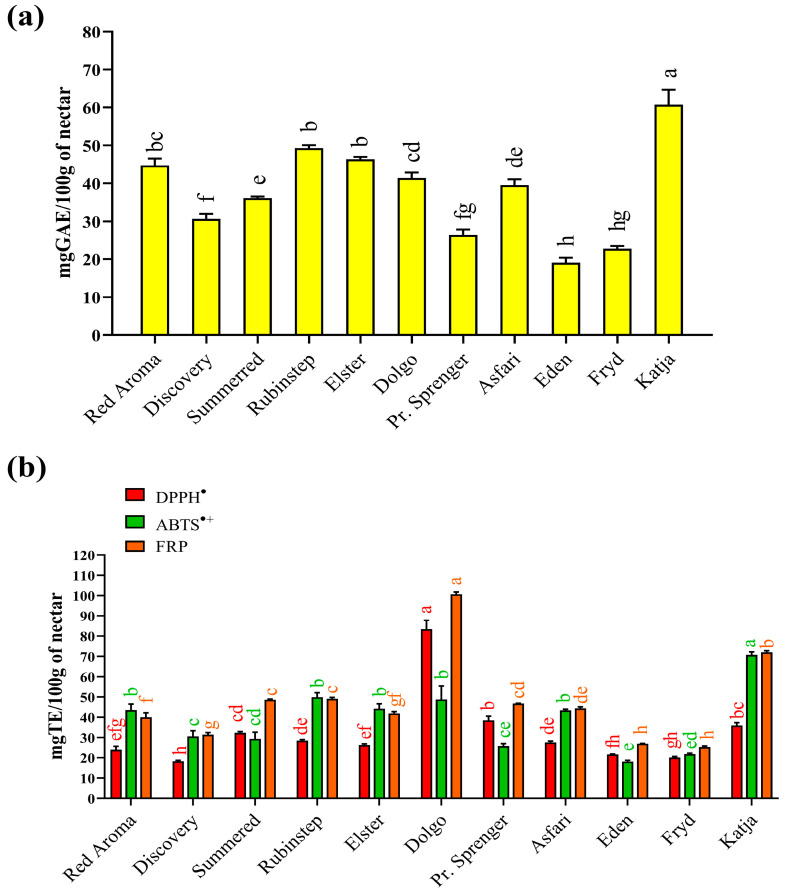
(**a**) Total phenolic content and (**b**) antioxidant properties of different apple nectar samples. Values are presented as means ± standard deviations (mean ± SD). The same coloured bars followed by the same coloured lower case letters are not significantly different (*p* < 0.05), according to Tukey’s test. **Abbreviations:** TE—Trolox; GAE—Gallic acid equivalent; ABTS^•+^—ABTS^•+^ scavenging activity; DPPH^•^—DPPH^•^ scavenging activity; FRP—Ferric-reducing power.

Results for total phenolic content (TPC) and antioxidant activity measured by FRP, ABTS^•+^, and DPPH^•^ assays are shown in Figure 1. The TPC in the collected nectar samples (Figure 1a) varied significantly, ranging from 19.1 (‘Eden’) to 60.7 (‘Katja’) mg/100 g GAE FW depending on the apple varieties used. While the highest value was significantly different from all others, the result for ‘Eden’ nectar was not statistically significantly different from the TPC value determined for ‘Fryd’ nectar (22.7 mg/100 g GAE FW). There is little data in the literature on the total phenolic content of nectar. Comparable data were found only for honey samples obtained directly from açaí floral nectar in Brazil [66]. In that case, the authors reported significantly higher TPC values (79.5–291.8 mg equivalent/100 g), which may be related to the different geographical and botanical origins of the collected floral nectar samples. In Serbia, there are no data on TPC values for nectar. However, there is a report for honeydew [89], which can be considered similar. In that case, the TPC value varied in a similar way to the present study, with the lowest value (48 mg/100 g catechin equivalents) for oak honeydew from Kraljevo (central Serbia) and the highest value (135 mg/100 g catechin equivalents) for oak/plum honeydew from Prokuplje (southern Serbia).

Examination of antioxidant properties is a fundamental biological test to determine whether a natural product can help combat oxidative stress, particularly free radical activity. For this reason, the collected nectar samples were tested for their quenching ability using two standard assays: DPPH^•^ and ABTS^•+^ radical neutralisation. Based on the results obtained (Figure 1b), two nectar samples stood out as the best sources of antioxidants capable of quenching free radicals. The nectar sample from the ‘Katja’ variety (70.7 mg/100 g TE FW) showed the highest activity against ABTS radical cations, while the sample from the ‘Dolgo’ apple variety exhibited the highest ability to neutralise DPPH radicals (83.4 mg/100 g TE).

The result for ‘Katja’ nectar was expected, as it was consistent with the highest TPC value determined. In addition, the unique phenolic profile (Table 1) of ‘Katja’ nectar likely contributed to its ability to scavenge hydrophilic ABTS radical cations effectively. However, for DPPH^•^, the highest ability to neutralise this radical was observed in the nectar sample from the ‘Dolgo’ variety, which was not consistent with the TPC results. This radical is less hydrophilic and may react more readily with other, more hydrophobic antioxidants in the nectar. Other predominant constituents of nectar [16], especially amino acids, peptides, or proteins, could also contribute to its antioxidant activity. Different DPPH^•^ quenching abilities have also been reported in the literature for nectars obtained from açai in Brazil, with values varying between 114.1 and 396.5 µmol eq./100 mL [66].

Apart from their ability to quench radicals, the ferric-reducing power (FRP) assay enabled determination of the redox capability of the nectar’s antioxidants in relation to potentially toxic ions such as Fe^2+^. Samples of the ‘Dolgo’ (100.6 mg/100 g TE FW) and ‘Katja’ (72.1 mg/100 g TE FW) cultivars proved to be the most effective reducing agents. In addition to phenolic compounds, the reducing ability of nectar samples also depends on other constituents, such as reducing sugars, certain elements, and amino acids. Another limitation of spectrophotometric tests is the possibility of obtaining falsely high or low results, as some antioxidant compounds can absorb at the same wavelength [90], while others react too slowly with the reagents used to develop a colour during measurement [91]. Literature data for honeydew samples from Serbia [89] also confirmed the significant ability of the samples to react with ferric ions as measured by the FRAP test (490–3090 µmol Fe^2+^/100 g), with large differences between samples depending on their botanical origin.

### 3.4. Raman Spectroscopy as a Tool for Characterisation and Discrimination of Apple Nectar

The chemical identification of nectar is primarily based on its carbohydrate structures. Nectar is rich in mono- and disaccharides, especially sucrose, which is transported from phloem tissue to nectary glands, as well as its component hexoses, glucose and fructose, in varying proportions [92]. To a lesser extent, additional nectar constituents play various roles in the nutrition and health of pollinators or are associated with herbivore defence, such as polyphenols (e.g., coumarins), alkaloids, vitamins, organic acids, ions, hormones, amino acids, fatty acids, and volatile substances [31,36].

**Figure 2:**
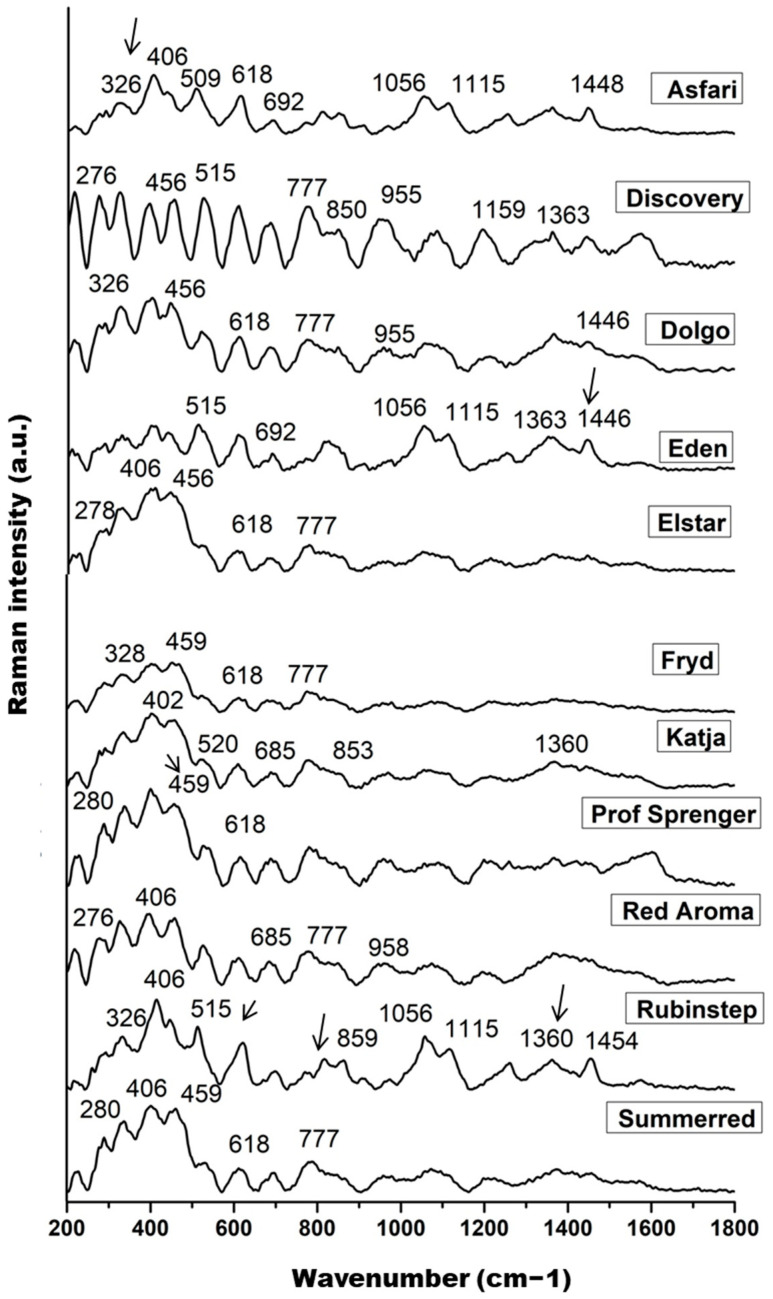
The pre-processed Raman spectra of nectar samples in the range of 200 to 1800 cm^−1^; band indicated on the glucose (326, 406, 456, 509–520, 777, and 850 cm^−1^), fructose (618, 1056, and 1446–1454 cm^−1^), and sucrose content (1360 and 1115 cm^−1^).

The measured Raman spectra of the nectar samples are shown in Figure 2, where each spectrum represents the mean of 10 spectra for each *Malus* sp. variety. The main features dominating the fingerprint region are signals at 326, ~406, 456, 515, 618, ~685, 777, and 850 cm^−1^, followed by bands around 955, 1056, 1115, 1159, 1363, and ~1446 cm^−1^, which can be identified as fingerprints of the main sugar components of nectar: fructose, glucose, and sucrose.

Skeletal vibrations dominate the range between 200 and 600 cm^−1^, with significant contributions from the main skeletal vibrations δ(C–C–C), δ(C–C–O), δ(C–O), and τ(C–C) of the saccharide molecules, primarily glucose and fructose [93]. The lower intensity band at approximately 326 cm^−1^ represents an endocyclic C–C–O and C–C–C ring mode of glucose [92]. The higher intensity band at 406 cm^−1^, its shoulder at 456 cm^−1^, and the bands in the range 509–520 cm^−1^ are probably due to the C–C–O bending vibration of α- and β-glucose and the C–C–C skeletal vibration of glucose, respectively. The band in the 515 cm^−1^ region is more pronounced in ‘Rubinstep’ and especially in ‘Discovery’, suggesting a higher glucose concentration. Bands around 520 cm^−1^ may also be attributed to fructose and sucrose [93]. The weak and medium strength bands at 777 and 850 cm^−1^ were assigned to the C–C stretching vibrations, C–H deformation, and C–C–O stretching and O–C–O bending vibrations of glucose [92,94].

The Raman bands at approximately 1360 and 1115 cm^−1^ can be assigned to C–OH deformations and the asymmetric and symmetric CH_2_ modes or OH bending vibrations of sucrose and their hydrolysed form, glucose, the most abundant saccharides in nectar samples [92]. These bands may indicate a higher concentration of glucose in ‘Discovery’ and ‘Rubinstep’.

The signals at 618 and 1056 cm^−1^ were clearly assigned to the fructose spectrum and associated with ring deformation of fructose [95,96]. The band in the range 1446–1454 cm^−1^ indicates CH_2_ vibrations that correlate with fructose [97]. The bands at 618, 1056, and ~1450 cm^−1^ are sharper and of higher intensity in ‘Discovery’ and ‘Rubinstep’, while in the others they appear with slightly lower intensity or are absent. These findings may indicate a higher concentration of fructose in ‘Discovery’ and ‘Rubinstep’. Moreover, according to some studies, vibrations in the same spectral region (940–1175 cm^−1^) may also be due to the C–OH group and the C–C and C–O stretches in the carbohydrate structure, as well as C–O in phenols [95].

### 3.5. Principal Component Analysis (PCA)

**Figure 3:**
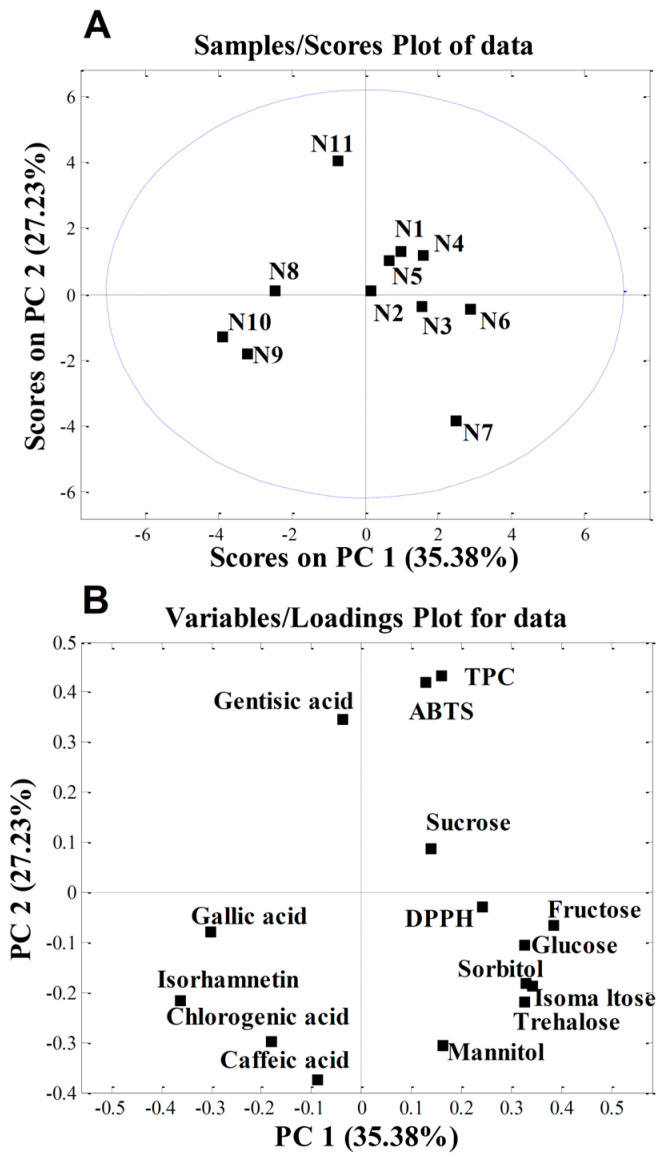
Principal component analysis (PCA) of nectar samples: (**A**) PC score plot; (**B**) loading plot. The abbreviations shown in Table 3: N1—‘Red Aroma’; N2—‘Discovery’; N3—‘Summerred’; N4—‘Rubinstep; N5—‘Elstar’; N6—‘Dolgo’; N7—‘Pr. Sprenger’; N8—‘Asfari’; N9—‘Eden’; N10—‘Fryd’; N11—‘Katja’.

PCA was applied to the quantitative data obtained in this study to determine whether the cultivar influenced the chemical composition of the apple samples investigated. The initial matrix comprised 11 samples and 15 variables (phenolic compounds, TPC, DPPH, ABTS, sugars, and sugar alcohols). PCA produced a two-component model explaining 62.61% of the total variance, with PC1 accounting for 35.38% and PC2 for 27.23%. The score plot in Figure 3A shows the separation of individual samples. According to the loading plot (Figure 3B), sample N11 (‘Katja’) was separated from the others due to its higher gentisic acid content and elevated TPC and ABTS values. These three traits are correlated, which was previously demonstrated by Sing et al. [98], who found that among the 12 phenolic acids tested, gentisic acid exhibited the highest ABTS activity.

The higher isorhamnetin content in samples N9 (‘Eden’) and N10 (‘Fryd’) accounted for their separation. We can speculate that, since these two cultivars have a very low level of sugars in their nectar, which is important for insect attraction, they are high in isorhamnetin, which acts as a UV-absorbing pigment. This creates a “nectar guide” in the centre of the flower that is visible to insects and directs them towards the nectar reward [99].

In contrast, sample N7 (‘Professor Sprenger’) was distinguished mainly by its higher content of the sugars trehalose and isomaltose and the sugar alcohol mannitol. According to von Firsch [100], sucrose, (iso)maltose, glucose, fructose, trehalose, and melesitose are sweet for bees; lactose, melibiose, raffinose, xylose, and arabinose are tasteless; mannose and galactose are toxic to bees; and gentibiose and cellobiose are repellent to bees. Since ‘Professor Sprenger’ had the highest levels of almost all sugar components, including trehalose, isomaltose, and mannitol, its nectar tastes the sweetest to bees. This is consistent with our previous studies, which showed that ‘Professor Sprenger’ is one of the best pollinisers for apple in intensive orchards [69].

**Figure 4:**
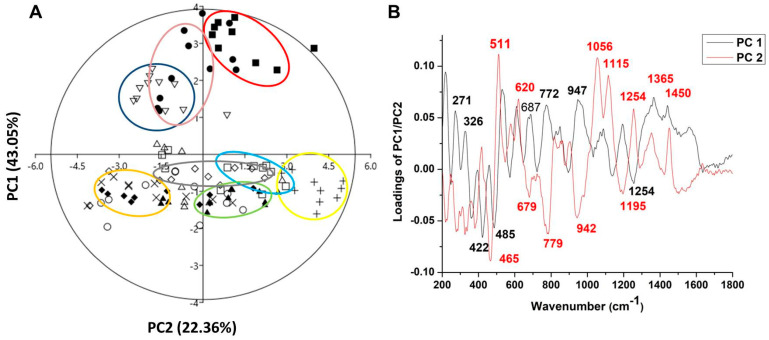
PCA applied to the data obtained from Raman spectra of nectar apple cultivars in the form of a score plot (**A**) and loading plot (**B**). Symbols indicate the different cultivars: closed circle—‘Asfari’, plus—‘Discovery’, open square—‘Dolgo’, closed square—‘Eden’, exes—‘Elstar’, open circle—‘Fryd’, open rhombus—‘Katja’, open triangle—‘Professor Sprenger’, closed triangle—‘Red Aroma’, open inverted triangle—‘Rubinstep’, and closed rhombus—‘Summerred’. The ellipses are a visual aid for grouping nectar samples belonging to the same cultivar, with no formal statistical significance.

The combination of Raman spectra of *Malus* sp. nectar samples with principal component analysis (PCA) provided a spectral fingerprint associated with the biochemical differences in eleven apple nectar samples. The PCA model for the nectar samples resulted in three principal components that explained 75.47% of the original Raman spectra variance (PC1—43.05%, PC2—22.36%, and PC3-10.06%), and loadings for the first two PCs are shown in Figure 4.

The score plot of PC1/PC2 (Figure 4A) suggests the existence of two groups of objects along the PC1 axis; for example, ‘Asfari’, ‘Eden’, and ‘Rubinstep’ differ from all other samples. The loading plot (Figure 4B) shows that the variables with a positive influence along the PC1 axis corresponded to the signals at 271, 326, 687, 772, 947, 1365, and 1450 cm^−1^, which have the greatest impact on the differences between ‘Discovery’, ‘Dolgo’, ‘Eden’, and ‘Red Aroma’ compared to all other samples. The highest positive-intensity loadings along PC1 at 271, 326, 772, and 1365 cm^−1^ are assigned to the C–C and C–H stretching vibrations in glucose and C-OH deformation present in glucose and sucrose [94], while the loading at 1450 cm^−1^, indicating CH_2_ vibration, is correlated with fructose [97]. According to these variables, we can suggest that ‘Discovery’, ‘Dolgo’, ‘Summerred’, and ‘Red Aroma’ may differ in nectar glucose and fructose content compared to ‘Eden’, which is indicated by the large distance between the mentioned clusters according to PC1. These differences suggest that ‘Eden’ and ‘Asfari’ are very close. On another hand, ‘Asfari’, ‘Summerred’, and ‘Rubinstep’ differ from others in the vibration of C-N from proteins and amino acids, according to negative loading at 1254 cm^−1^, and glucose [96].

According to PC2, nectar samples from ‘Asfari’, ‘Eden’, and ‘Rubinstep’ differ from the samples mainly in glucose content, as indicated by positive intensity loadings at 511 cm^−1^ from C-C-C skeletal bending vibration, and in fructose, related to bands at 620, 1056, and 1450 cm^−1^ (from ring deformation of fructose). To a lesser extent, differences are observed in sucrose, related to bands at 1365 and 1115 cm^−1^.

Negative loadings of PC2 indicate that ‘Elstar’, ‘Discovery’, ‘Dolgo’, ‘Fryd’, ‘Katja’, and ‘Professor Sprenger’ differ from the other samples mainly in the band at 465 cm^−1^, which is related to the stretching and bending vibrations of C-O, C-C-O, and C-C-C that form the molecular structure of sugars, as well as the stretching vibrations of unsaturated rings found in flavones, flavonoids, and polyphenols, located at 679 cm^−1^ [101]. To a lesser extent, these differences occur in glucose (the band at 779 cm^−1^ appears due to the deformation of C-H), sucrose (the band at 942 cm^−1^ is characteristic of deformation vibrations of C-H and methylene bonds—CH_2_, as well as the bending vibrations of C-O-H) and proteins and amino acids (1195 cm^−1^ is associated with the C-N bonds of proteins and amino acids) [102].

In this study, it has been demonstrated that the Raman technique is an analytical tool with advantages for the analysis of nectar, as it is environmentally friendly and easy to use, does not require chemicals for analysis, and provides results in less time. This technique can discriminate between different apple nectar samples based on sucrose, glucose, and fructose content, as well as polyphenols and proteins. The results of Raman spectroscopy showed that the nectar of the ‘Red Aroma’, ‘Discovery’, and ‘Summerred’ differ in glucose and/or fructose concentration from that of ‘Eden’ and ‘Asfari’. The grouping of ‘Eden’, ‘Asfari’, and ‘Rubinstep’ indicated some similarities, mainly based on glucose content. In terms of PCA, the nectar samples of ‘Elstar’, ‘Dolgo’, ‘Fryd’, and ‘Katja’ differ from the other samples mainly in sugar, polyphenol, and protein concentration.

## 4. Conclusions

According to this study, significant differences were observed among the apple cultivars studied, regardless of whether they belonged to *M. domestica* or *M. sylvestris*. Although all examined apple cultivars had glucose, sucrose, and fructose as the dominant sugars in their nectar, cultivars ‘Rubinstep’, ‘Dolgo’, and ‘Professor Sprenger’ had the highest levels of each sugar component and total sugars. ‘Eden’ and ‘Fryd’ had the highest level of isorhamnetin, while ‘Dolgo’ exhibited the highest ability to neutralise DPPH radicals and proved to be the best reducing agent. The chemical content of apple nectar has shown that it is an excellent source of bioactive compounds that can positively impact human health by enriching the diet with numerous phenolics, thus qualifying it as a “superfood”.

However, we did not conduct any bee counts during nectar collection; therefore, it is unclear whether these differences influenced bee activity. However, we can speculate that sugar levels, which are very important for bee foraging, made some cultivars more interesting to insects and are, thus, better pollinisers, as demonstrated in our previous studies [45,69]. Conversely, cultivars with low sugar levels (‘Eden’ and ‘Fryd’), which our previous studies identified as “weak” pollenisers [95], also had high levels of phenolic compounds, especially isorhamnetin. Whether isorhamnetin can be considered a deterrent chemical for insect pollenisers will be answered by future studies.

## Figures and Tables

**Table 1:** Apple cultivars used in this study.

Cultivar	Species	Main Cultivar or Polliniser	Ripening Time	Yields	Code
‘Red Aroma’	*M. domestica*	Main cultivar	Late	High	N1
‘Discovery’	*M. domestica*	Main cultivar	Early	Average	N2
‘Summerred’	*M. domestica*	Main cultivar	Mid	High	N3
‘Rubinstep’	*M. domestica*	Main cultivar	Late	High	N4
‘Elstar’	*M. domestica*	Main cultivar	Late	High	N5
‘Dolgo’ (crab apple)	*M. sylvestris*	Polliniser	Mid	Low	N6
‘Professor Sprenger’ (crab apple)	*M. sylvestris*	Polliniser	Late	Low	N7
‘Asfari’	*M. domestica*	Main cultivar	Mid	Average	N8
‘Eden’	*M. domestica*	Main cultivar	Mid	Average	N9
‘Fryd’	*M. domestica*	Main cultivar	Late	Average	N10
‘Katja’	*M. domestica*	Polliniser	Mid	Average	N11

**Table 2:** Contents of sugars and sugar alcohols in analysed nectar samples (g/100 g).

Sort	Sorbitol	Mannitol	Trehalose	Glucose	Fructose	Sucrose	Isomaltose	Sum
‘Red Aroma’	0.56 ± 0.02 b *	0.240 ± 0.004 c	0.61 ± 0.03 b	7.3 ± 0.3 c	15 ± 2 d	5.4 ± 0.3 e	2.9 ± 0.2 b	31.6 d
‘Discovery’	0.73 ± 0.02 c	0.460 ± 0.007 d	0.59 ± 0.03 b	13.4 ± 0.6 e	19 ± 2 f	0.29 ± 0.02 ab	0.34 ± 0.03 a	35.1 e
‘Summerred’	1.55 ± 0.04 d	0.190 ± 0.003 bc	2.6 ± 0.2 e	10.8 ± 0.5 d	19 ± 2 ef	0.84 ± 0.05	3.9 ± 0.3 c	38.5 f
‘Rubinstep’	0.72 ± 0.02 c	0.140 ± 0.002 b	1.67 ± 0.08 c	19.5 ± 0.9 g	25 ± 3 g	0.48 ± 0.03 b	0.21 ± 0.02 a	47.3 g
‘Elstar’	0.70 ± 0.02 c	0.210 ± 0.003 c	3.0 ± 0.2 f	5.6 ± 0.3 b	12 ± 1 c	1.20 ± 0.08 c	0.9 ± 0.1 a	23.1 c
‘Dolgo’	3.49 ± 0.08 f	-	2.2 ± 0.1 d	8.0 ± 0.4 c	17 ± 2 e	2.2 ± 0.1 d	5.1 ± 0.4 e	38.3 f
‘Professor Sprenger’	2.29 ± 0.05 e	2.88 ± 0.05 d	4.5 ± 0.2 f	15.6 ± 0.7 f	21 ± 2 f	0.50 ± 0.03 b	4.2 ± 0.3 d	50.8 g
‘Asfari’	0.070 ± 0.001 a	0.040 ± 0.001 a	0.080 ± 0.004 a	0.56 ± 0.03 a	1.2 ± 0.1 ab	1.47 ± 0.09 c	-	3.4 b
‘Eden’	0.040 ± 0.001 a	0.240 ± 0.004 c	0.110 ± 0.005 a	0.190 ± 0.008 a	0.33 ± 0.04 a	0.130 ± 0.008 a	-	1.0 a
‘Fryd’	-	-	-	0.49 ± 0.03 a	0.21 ± 0.03 a	0.010 ± 0.001 a	-	0.7 a
‘Katja’	0.060 ± 0.001 a	0.110 ± 0.002 b	-	0.100 ± 0.007 a	2.4 ± 0.3 b	0.030 ± 0.002 a	-	2.7 ab

* Different letters within the same column indicate statistically significant differences at *p* < 0.05 via Tuckey’s test.

**Table 3:** Identification and characterisation of phenolic compounds in nectar samples of different apple varieties by UHPLC Q-ToF MS (untargeted analysis). The identified compounds, retention time (RT), molecular formula, calculated and exact mass, and MS fragments are listed in Table 1.

No.	RT	Compound Name	Formula	Calculated Mass	*m/z* Exact Mass	mDa	MS Fragments	Presence of Compounds in Nectar Samples
***Phenolic acids and derivatives***								
*Hydroxybenzoic acid and derivatives*								
**1**	5.66	**Hydroxybenzoic acid**	C_7_H_5_O_3_^−^	137.0239	137.0220	1.85	**108.0180(100)**	**N1-5,N7-11**
**2**	6.53	**Hydroxyphenylacetic acid**	C_8_H_7_O_3_^−^	151.0395	151.0368	2.71	**107.0468(100)**, 108.0191(59)	**N11**
**3**	3.30	**Hydroxybenzoic acid hexoside is. I**	C_13_H_15_O_8_^−^	299.0767	299.0728	3.91	**137.0212(100)**	**N4**
**4**	5.73	**Hydroxybenzoic acid hexoside is. II**	C_13_H_15_O_8_^−^	299.0767	299.0742	2.54	**137.0215(100)**, 136.0118(35)	**N1,N2,N4,N5,N7,N8**
**5**	3.30	**Dihydroxybenzoic acid** (like gentisic acid) *	C_7_H_5_O_4_^−^	153.0188	153.01645	2.34	**108.0188(100)**, 109.0262(81)	**N1-3,N5-8, N11**
**6**	5.93	**Dihydroxybenzoic acid pentosyl hexoside**	C_18_H_23_O_13_^−^	447.1144	447.1154	−0.94	**152.0083(100)**, 108.0192(14), 109.0258(13), 153.0137(14), **315.0678(2)**, 447.11(36)	**N2,N3,N11**
**7**	1.55	Gallic acid *	C_7_H_5_O_5_^−^	169.0137	169.0112	2.46	**125.021(100)**, 124.0132(70)	**N1,N2,N5,N7,N8,N10**
**8**	5.85	**Methyl gallate**	C_8_H_7_O_5_^−^	183.0293	183.0262	3.07	**124.0128(100)**, 123.0053(54), 168.0004(2)	**N8**
**9**	7.68	**Ethyl gallate**	C_9_H_9_O_5_^−^	197.0450	197.0422	2.85	**124.0134(100)**, 125.0201(27), 169.0098(2)	**N5,N7-11**
**10**	3.70	**Vanillyl alcohol**	C_8_H_9_O_3_^−^	153.0552	153.0519	3.25	**123.0420(100)**, 122.0342(75)	**N1-5,N8-10**
**11**	4.44	**Vanillic acid hexoside**	C_14_H_17_O_9_^−^	329.0873	329.0849	2.38	**121.0259(100)**, 123.0402(2), 152.0087(2), 167.0278(1)	**N1-9,N11**
*Hydroxycinnamic acid and derivatives*								
**12**	6.66	**Coumaric acid hexoside**	C_15_H_17_O_8_^−^	325.0923	325.0884	3.89	**119.0472(100)**, 163.0367(20)	**N5,N8,N10**
**13**	7.68	**Coumaroylquinic acid**	C_16_H_17_O_8_^−^	337.0923	337.0883	4.03	**191.0523(100)**, 119.0474(2), 127.0370(3), **163.0371(1)**, 173.0414(2)	**N1-3,N5-10**
**14**	6.86	Caffeic acid *	C_9_H_7_O_4_^−^	179.0344	179.0339	0.46	**135.0416(100)**, 134.0335(72)	**N1-3,N5,N7-11**
**15**	10.04	**Ethyl caffeic acid (ethyl caffeate)**	C_11_H_11_O_4_^−^	207.0657	207.0628	2.92	**135.0399(100)**, 133.0254(82), 134.0308(36), 161.0149(10)	**N1**
**16**	6.60	Caffeoylquinic acid (like chlorogenic acid) *	C_16_H_17_O_9_^−^	353.0873	353.0842	3.09	**191.0523(100)**, 135.0407(2), 161.0204(2), 173.0409(1), 179.0326(1)	**N1-3,N6-10**
**17**	7.41	**Ferulic acid hexoside**	C_16_H_19_O_9_^−^	355.1029	355.0974	5.45	**134.0343(100)**, 149.0571(30), 160.0118(60), 175.0357(68), 193.0462(38)	**N2,N3,N5,N9,N10**
**18**	7.48	**Feruloylquinic acid**	C_17_H_19_O_9_^−^	367.1029	367.0991	3.83	**191.0523 (100)**, 111.0421(11), 134.0345(30), 149.0581(2), 155.0320(2), 173.0415(10), 193.0481(17)	**N1,N2,N6-11**
**19**	9.70	**Feruloyl-caffeoylquinic acid**	C_26_H_25_O_12_^−^	529.1352	529.1324	2.72	**127.0372(100)**, 153.0153(83), 161.0242(5), 173.0414(4), 179.0309(5), 191.0522(24), 529.1478(7)	**N3**
*Hydroxycinnamic acid amides (Phenylamides)*								
**20**	4.17	**Coumaroyl putrescine** **(*N*^1^-coumaroyl putrescine)**	C_13_H_19_N_2_O_2_^+^	235.1447	235.1443	0.43	**119.0493(100)**, 147.0437(99)	**N1-5,N7-10**
**21**	8.82	**Dicoumaroyl putrescine** **(*N*^1^*,N*^6^-di-coumaroyl putrescine)**	C_22_H_25_N_2_O_4_^+^	381.1814	381.1845	−3.08	**147.0438(100)**, 119.0495(10), 218.1168(5), 235.1438(4)	**N1,N5-11**
**22**	7.81	**Dicoumaroyl spermidine** **(*N*^1^*,N*^5^-di-coumaroyl spermidine)**	C_25_H_32_N_3_O_4_^+^	438.2393	438.2387	0.63	**147.0438(100)**, 119.0495(6), 204.1014(57), 218.1165(16), 275.1750(16), 292.2013(47), 421.2144(11)	**N1-5,N7,N9-11**
**23**	10.10	**Tricoumaroyl spermidine** **(*N*^1^*,N*^5^*,N*^10^-tri-coumaroyl spermidine)**	C_34_H_38_N_3_O_6_^+^	584.2755	584.2802	−4.65	**438.2389(100)**, 147.0442(43), 204.1016(49), 218.1178(5), 275.1752(15), 292.2015(23), 420.2277(26)	**N1-11**
**24**	9.76	**Dicoumaroyl caffeoyl spermidine** **(*N*^1^*,N*^5^-di-coumaroyl-*N*^10^-caffeoyl spermidine)**	C_34_H_38_N_3_O_7_^+^	600.2711	600.2751	−4.06	**454.2333(100)**, 147.0439(42), 204.1018(48), 220.0960(14), 275.1748(15), 292.1998(30), 438.23783(61),	**N1,N2,N4,N5,N8,N9**
*Coumarins and derivatives*								
**25**	6.79	**Aesculetin**	C_9_H_5_O_4_^−^	177.0193	177.0181	1.21	**105.0319(100)**, 121.0264(28), 133.0262(62), 149.0209(27)	**N1-5,N7-11**
**26**	6.53	**Aesculin**	C_15_H_15_O_9_^−^	339.0716	339.0678	3.79	**177.0161(100)**, 133.0256(6)	**N5**
***Flavonoids***								
*Flavonol aglycones and glycosides*								
**27**	10.71	**Kaempferol**	C_15_H_9_O_6_^−^	285.0399	285.0403	−0.41	**285.0359(100)**, 143.047(9), 159.0412(11), 171.0415(6), 187.0361(14), 211.0344(9), 229.0460(12), 239.0299(11)	**N9,N10**
**28**	10.97	**Kaempferide**	C_16_H_11_O_6_^−^	299.0556	299.0503	5.32	**255.0262(100),** 211.0364(3), 227.0311(80), 228.0341(13), 284.0286(23), 285.0317(5)	**N7,N8**
**29**	8.89	**Kaempferol 3-*O*-pentoside**	C_20_H_17_O_10_^−^	417.0822	417.0817	0.53	**284.0284(100)**, 227.0315(30), 255.0258(53), 285.0356(51)	**N1,N4,N9,N10**
**30**	9.03	**Kaempferol 3-*O*-rhamnoside**	C_21_H_19_O_10_^−^	431.0978	431.0963	1.46	**284.0288(100)**, 227.0309(18), 255.0262(34), 285.0357(95)	**N1-11**
**31**	10.98	**Quercetin-dimethyl-ether**	C_17_H_13_O_7_^−^	329.0661	329.0619	4.23	211.1321(11), 215.0294(9), 243.0254(9), 255.0243(5), 271.021(57), **299.0149(100)**, 300.0187(20)	**N2,N4,N5,N7-10**
**32**	8.49	**Quercetin 3-*O*-pentoside**	C_20_H_17_O_11_^−^	433.0771	433.0776	−0.50	**301.0289(100)**, 150.9987(16), 178.9952(19), 300.0242(79)	**N10**
**33**	8.56	**Quercetin 3-*O*-rhamnoside**	C_21_H_19_O_11_^−^	447.0933	447.0931	0.14	**300.0234(100)**, 151.0003(8), 178.9960(6), 271.0205(15), 301.0301(68)	**N3,N7,N8,N10,N11**
**34**	10.64	Isorhamnetin *	C_16_H_11_O_7_^−^	315.0505	315.0487	1.79	**300.0238(100)**, 137.9923(22), 165.9874(49), 174.0287(25), 243.0261(23), 255.0257(33), 271.0204(21), 301.0269(22)	**N1,N2,N4,N5,N7-11**
**35**	8.43	**Isorhamnetin 3-*O*-hexoside**	C_22_H_21_O_12_^−^	477.1033	477.1086	−5.35	**299.0155(100)**, 271.0204(20), 300.0212(45), 314.0384(45), **315.0458(18)**, 477.0987(6)	**N2,N10**
**36**	7.95	**Isorhamnetin 3-*O*-(2″-*O*-rhamnosyl)hexoside**	C_28_H_31_O_16_^−^	623.1618	623.1596	2.18	**314.0389(100)**, 271.0202(5), 299.0154(43), 300.0202(11), 315.0438(29), **459.0897(3)**	**N1,N2,N4,N5,N7,N9,N10**
**37**	7.74	**Isorhamnetin 3-*O*-(2″-*O*-hexosyl)hexoside**	C_28_H_31_O_17_^−^	639.1561	639.1533	2.82	**314.0391(100)**, 271.0204(6), 299.0156 (46), 300.0217(20), 315.0445(44), **459.0921(5)**, 639.1508(53)	**N1,N2,N4,N5,N7-10**
**38**	8.01	**Isorhamnetin 3-*O*-(2″-hexosyl-6″-maloyl) hexoside**	C_31_H_33_O_20_^−^	725.1565	725.1596	−3.13	**681.1621(100)**, 299.0144(30), 300.0212(12), 314.0391(86), 315.0432(31), **501.1024(4)**	**N1,N2,N4,N5,N10**
**39**	10.78	**Syringetin**	C_17_H_13_O_8_^−^	345.0616	345.0569	4.62	**330.0342(100)**, 149.0209(19), 243.0259(22), 259.0211(15), 271.0206(18), 287.0159(18), **315.0107(78)**	**N1,N2,N4,N5,N7-11**
**40**	8.55	**Syringetin 3-*O*-hexoside**	C_23_H_23_O_13_^−^	507.1139	507.1136	0.28	**329.0260(100)**, 301.0314(17), 330.0302(27), 344.0484(29), 345.0542(10), 507.1101(11)	**N2,N4,N10**
**41**	8.01	**Syringetin 3-*O*-(2″-*O*-rhamnosyl)hexoside**	C_29_H_33_O_17_^−^	653.1718	653.1654	6.40	**344.0494(100)**, 329.0261(51), 330.0302(13), 345.0542(34), **489.0944(2)**, 653.1669(48)	**N1,N2,N4,N5,N7-11**
*Other detected flavonoids*								
**42**	9.70	**Eriodictyol**	C_15_H_11_O_6_^−^	287.0556	287.0522	3.36	**135.042(100)**, 107.0107(17), 123.0415(41), 125.0208(21), 150.9999(18), 167.0315(36)	**N2,N7-9**
**43**	7.75	**Taxifolin**	C_15_H_11_O_7_^−^	303.0505	303.0487	1.77	**230.0182(100)**, 149.0572(8), 175.036(10), 186.0286(15), 189.0518(23), 213.0522(10), 231.022(18), 241.0465(9)	**N2-5,N7-11**
*Dihydrochalcone glycosides*								
**44**	8.96	**Phloretin 2′-*O*-hexoside (Phlorizin)**	C_21_H_23_O_10_^−^	435.1291	435.1258	3.28	**167.0323(100)**, 123.0401(23), 125.0208(28), 273.0724(45)	**N7**
**45**	8.29	**Phloretin 2′-*O*-(6″-*O*-hexosyl)hexoside**	C_27_H_33_O_15_^−^	597.1819	597.1757	6.18	**273.0727(100)**, 123.0421(2), 125.0209(6), 167.0313(24), 168.0350(2), 597.1803(4)	**N7**
***Organic acid and derivatives***								
**46**	0.87	**Citric acid**	C_6_H_7_O_7_^−^	191.0192	191.0165	2.74	**111.0059(100)**, 112.0091(5)	**N1-11**
**47**	5.99	**Isopropylmalic acid**	C_7_H_11_O_5_^−^	175.0606	175.0582	2.39	**115.0373(100)**, 113.0581(53), 131.0681(3)	**N1-11**

Abbreviations: Nectar samples: N1—‘Red Aroma’; N2—‘Discovery’; N3—‘Summerred’; N4—‘Rubinstep’; N5—‘Elstar’; N6—‘Dolgo’; N7—‘Pr. Sprenger’; N8—‘Asfari’; N9—‘Eden’; N10—‘Fryd’; N11—‘Katja’. * Compounds confirmed based on available standards.

**Table 4:** Content (mg/kg) of some detected phenolic compounds in apple nectar samples, using available standards.

Phenolic Compounds	N1	N2	N3	N4	N5	N6	N7	N8	N9	N10	N11
	(mg/kg Nectar)										
**Dihydroxybenzoic acid (Gentisic acid)**	1.75	4.50	6.35	-	1.48	<LOQ	<LOQ	3.18	-	-	39.06
**Gallic acid**	<LOQ	<LOQ	-	-	<LOQ	-	<LOQ	1.22	-	1.53	-
**Caffeic acid**	<LOQ	<LOQ	<LOQ	-	<LOQ	-	2.54	1.24	1.15	0.87	<LOQ
**Caffeoylquinic acid (Chlorogenic acid)**	0.67	2.04	3.32	-	-	7.09	3.67	7.20	8.59	5.42	-
**Isorhamnetin**	1.34	4.92	-	1.72	5.43	-	12.52	15.63	49.04	50.83	6.46

**Abbreviations: Nectar samples: N1—‘Red Aroma’; N2—‘Discovery’; N3—‘Summerred’; N4—‘Rubinstep; N5—‘Elstar’; N6—‘Dolgo’; N7—‘Pr. Sprenger’; N8—‘Asfari’; N9—‘Eden’; N10—‘Fryd’; N11—‘Katja’.** Quantification using available standards. <LOQ—less then limit of quantification; “-” means nonidentified compounds in sample.

## Data Availability

Data are contained within the article.

## Supplementary Materials

The following supporting information can be downloaded at: https://www.mdpi.com/article/10.3390/antiox15010103/s1, Table S1: Equation parameters and correlation coefficient (R^2^) of used phenolic standards for quantification. Table S2: The presence of phenolic compounds in the nectar samples
