# Supplementary material for: Chemical Fingerprint of Floral Nectar in Apple (Malus sp.) Cultivars Grown in Norway
Source: Antioxidants (Basel). 2026 Jan 13;15(1):103. doi: 10.3390/antiox15010103 (PMC12837190; doi:10.3390/antiox15010103)
Supplement: Supplementary file 1 [file antioxidants-15-00103-s001.zip › antioxidants-4055174-supplementary.pdf]

Supplementary Materials: The following supporting information can be downloaded at: https://www.mdpi.com/article/doi/s1,

Table S1: Equation parameters and correlation coefficient (R^2) of used phenolic standards for quantification

Table S2: The presence of phenolic compounds in the nectar samples

Table S1. Equation parameters and correlation coefficient (R^2) of used phenolic standards for quantification

Standards	Y=a * X±b	R^2	Linear range (ppm)	LOD (ppm)	LOQ (ppm)
**Gentisic acid**	y= 1875463.9259 * x + 134879.1742	0.9972	0.1-7.0	0.46	1.52
**Chlorogenic acid**	y= 1214704.8084 * x + 12291.5523	0.9995	0.1-4.0	0.11	0.36
**Caffeic acid**	y= 3816298.5578 * x + 506521.1168	0.9916	0.1-3.0	0.35	1.17
**Gallic acid**	y= 4582773.7482 * x - 211812.5731	0.9989	0.1-10	0.46	1.52
**Isorhamnetin**	y= 1095743.6032 * x + 839703.9932	0.9995	0.1-9.0	0.35	1.17

Table S2. The presence of phenolic compounds in the nectar samples.

No	Compounds	Nectar samples										
		N1	N2	N3	N4	N5	N6	N7	N8	N9	N10	N11
Phenolic acids and derivatives												
Hidroxybenzoic acid and derivatives												
1	**Hydroxybenzoic acid**	+	+	+	+	+	-	+	+	+	+	+
2	**Hydroxy-phenylacetic acid**	-	-	-	-	-	-	-	-	-	-	+
3	**Hydroxybenzoic acid hexoside isomer I**	-	-	-	+	-	-	-	-	-	-	-
4	**Hydroxybenzoic acid hexoside isomer II**	+	+	-	+	+	-	+	+	-	-	-
5	**Dihydroxybenzoic acid (like gentisic acid)**	+	+	+	-	+	+	+	+	-	-	+
6	**Dihydroxybenzoic acid pentosyl hexoside**	-	+	+	-	-	-	-	-	-	-	+
7	**Gallic acid**	+	+	-	-	+	-	+	+	-	+	-
8	**Methyl gallate**	-	-	-	-	-	-	-	+	-	-	-
9	**Ethyl gallate**	-	-	-	-	+	-	+	+	+	+	+
10	**Vanillyl alcohol**	+	+	+	+	+	-	-	+	+	+	-
11	**Vanillic acid hexoside**	+	+	+	+	+	+	+	+	+	-	+
Hydroxycinnamic acid and derivatives												
12	**Coumaric acid hexoside**	-	-	-	-	+	-	-	+	-	+	-
13	**Coumaroylquinic acid**	+	+	+	-	+	+	+	+	+	+	-
14	**Caffeic acid**	+	+	+	-	+	-	+	+	+	+	+

**15**	**Ethyl caffeic acid**	+	-	-	-	-	-	-	-	-	-	-
**16**	**Caffeoylquinic acid (like chlorogenic acid)**	+	+	+	-	-	+	+	+	+	+	-
**17**	**Ferulic acid hexoside**	-	+	+	-	+	-	-	-	+	+	-
**18**	**Feruloylquinic acid**	+	+	-	-	-	+	+	+	+	+	+
**19**	**Feruloyl-caffeoylquinic acid**	-	-	+	-	-	-	-	-	-	-	-
	Hydroxycinnamic acid amides											
**20**	**Coumaroyl putrescine (*N*^{1}-coumaroyl putrescine)**	+	+	+	+	+	-	+	+	+	+	-
**21**	**Dicoumaroyl putrescine (*N*^{1},*N*^{6}-di-coumaroyl putrescine)**	+	-	-	-	+	+	+	+	+	+	+
**22**	**Dicoumaroyl spermidine (*N*^{1},*N*^{5}-di-coumaroyl spermidine)**	+	+	+	+	+	-	+	-	+	+	+
**23**	**Tricoumaroyl spermidine (*N*^{1},*N*^{5},*N*^{10}-tri-coumaroyl spermidine)**	+	+	+	+	+	+	+	+	+	+	+
**24**	**Dicoumaroyl caffeoyl spermidine (*N*^{1},*N*^{5}-di-coumaroyl-*N*^{10}-caffeoyl spermidine)**	+	+	-	+	+	-	-	+	+	-	-
	Coumarins and derivatives											
**25**	**Aesculetin**	+	+	+	+	+	-	+	+	+	+	+
**26**	**Aesculin**	-	-	-	-	+	-	-	-	-	-	-
	Flavonoids											
	Flavonol aglycones and glycosides											
**27**	**Kaempferol**	-	-	-	-	-	-	-	-	+	+	-
**28**	**Kaempferide**	-	-	-	-	-	-	+	+	-	-	-
**29**	**Kaempferol 3-*O*-pentoside**	+	-	-	+	-	-	-	-	+	+	-
**30**	**Kaempferol 3-*O*-rhamnoside**	+	+	+	+	+	+	+	+	+	+	+
**31**	**Quercetin-dimethyl-ether**	-	+	-	+	+	-	+	+	+	+	-
**32**	**Quercetin 3-*O*-pentoside**	-	-	-	-	-	-	-	-	-	+	-
**33**	**Quercetin 3-*O*-rhamnoside**	-	-	+	-	-	-	+	+	-	+	+
**34**	**Isorhamnetin***	+	+	-	+	+	-	+	+	+	+	+
**35**	**Isorhamnetin 3-*O*-hexoside**	-	+	-	-	-	-	-	-	-	+	-
**36**	**Isorhamnetin 3-*O*-(2''-*O*-rhamnosyl)hexoside**	+	+	-	+	+	-	+	-	+	+	-
**37**	**Isorhamnetin 3-*O*-(2''-*O*-hexosyl)hexoside**	+	+	-	+	+	-	+	+	+	+	-
**38**	**Isorhamnetin 3-*O*-(6''-*O*-hexosyl)-malonyl-hexoside**	+	+	-	+	+	-	-	-	-	+	-
**39**	**Syringetin**	+	+	-	+	+	-	+	+	+	+	+
**40**	**Syringetin 3-*O*-hexoside**	-	+	-	+	-	-	-	-	-	+	-
**41**	**Syringetin 3-*O*-(2''-*O*-rhamnosyl)hexoside**	+	+	-	+	+	-	+	+	+	+	+
	Other detected flavonoids											
**42**	**Eriodictyol**	-	+	-	-	-	-	+	+	+	-	-

**43**	**Taxifolin**	-	+	+	+	+	-	+	+	+	+	+
	Dihydrochalcone glycosides											
**44**	**Phloretin 2'-*O*-hexoside (Phlorizin)**	-	-	-	-	-	-	+	-	-	-	-
**45**	**Phloretin 2'-*O*-(6"-*O*-hexosyl)hexoside**	-	-	-	-	-	-	+	-	-	-	-
	Organic acid and derivatives											
**46**	**Citric acid**	+	+	+	+	+	+	+	+	+	+	+
**47**	**Isopropylmalic acid**	+	+	+	+	+	+	+	+	+	+	+

Abbreviations: Nectar samples: N1-Red Aroma; N2-Discovery; N3-Summered; N4-Rubinstep; N5-Elster; N6-Dolgo; N7-Pr. Sprenger; N8-Asfari; N9-Eden; N10-Fryd; N11-Katja. „+“ identified compounds; „–“ nonidentified compounds in apple nectar sample.
